# Adherence to Clinical Practice Guideline Recommendations in Women with Gestational Diabetes and Associations with Maternal and Infant Health—A Cohort Study

**DOI:** 10.3390/nu14061274

**Published:** 2022-03-17

**Authors:** Sara T. Mustafa, Jane E. Harding, Clare R. Wall, Caroline A. Crowther

**Affiliations:** 1 Liggins Institute, The University of Auckland, Grafton, Auckland 1023, New Zealand; sara.mustafa@auckland.ac.nz (S.T.M.); j.harding@auckland.ac.nz (J.E.H.); 2 Faculty of Medical and Health Sciences, The University of Auckland, Grafton, Auckland 1023, New Zealand; c.wall@auckland.ac.nz

**Keywords:** nutrition guidelines, pregnancy-induced diabetes, diet therapy, maternal health, infant health

## Abstract

Gestational diabetes mellitus (GDM) is managed by dietary advice, but limited evidence exists about the impact of adherence on health. We assessed whether adherence to the New Zealand Ministry of Health dietary recommendations is associated with maternal and infant health in women with GDM. Data from 313 women with GDM were used. Adherence to food-related recommendations was scored from 0 (no adherence) to 10 (adhered to all recommendations) and analysed in tertile groups (high, moderate, low adherence). Adherence to visiting a dietitian and appropriate weight gain were assessed as yes or no. Chi-square, ANOVA, and odds ratios were used to compare groups. High dietary adherence compared to low adherence was associated with reduced oral hypoglycaemic and insulin use (OR = 0.55, CI = 0.30–1.00). Visiting a dietitian compared to not was associated with increased oral hypoglycaemic and insulin use (OR = 2.96, CI = 1.12–7.80), decreased odds of a large-for-gestational-age infant (OR = 0.32, CI = 0.14–0.73) and neonatal hyperbilirubinaemia (OR = 0.27, CI = 0.08–0.95). Greater than recommended compared with recommended weight gain was associated with increased oral hypoglycaemic and insulin use (OR = 2.51, CI = 1.26–5.01), while lower than recommended weight gain was associated with decreased postpartum haemorrhage (OR = 0.45, CI = 0.23–0.91) and increased breastfeeding (OR = 1.96, CI = 1.04–3.70). Adherence to dietary recommendations for women with GDM likely improves health outcomes.

## 1. Introduction

Gestational diabetes mellitus (GDM) is characterised by elevated maternal blood glucose concentrations first identified mid-pregnancy. The primary management for GDM is dietary intervention to control blood glucose concentrations, with the addition of oral hypoglycaemics and insulin if needed [1]. Uncontrolled GDM is associated with an increased risk of adverse health outcomes in the mother and infant [2].

As with all treatment, adherence to the advice given is key [3]. Among women with GDM, moderate adherence has been reported for the dietary and pharmacological recommendations, including oral hypoglycaemics and insulin [4,5]. Some studies have found an association between achieving the appropriate gestational weight gain and high adherence to pharmacological guidelines for the management of GDM and improved pregnancy health outcomes [5,6]. Infants of women with high adherence to pharmacological treatments for GDM had lower rates of neonatal hypoglycaemia compared to infants of women with low adherence [5]. Good adherence to gestational weight gain recommendations among women with GDM has been associated with decreased risk of caesarean section, an infant born large-for-gestational-age (LGA), macrosomia, and neonatal hypoglycaemia [6]. However, there is limited information on the influence of maternal adherence to dietary guideline recommendations on maternal and infant health outcomes.

This study aims to identify whether maternal adherence to dietary guideline recommendations is associated with the health of mothers with GDM and their infants.

## 2. Materials and Methods

A cohort study nested within the Target Trial [7] was conducted to investigate dietary adherence in women with GDM in New Zealand. Women were diagnosed with GDM based on the results from a 75 g oral glucose tolerance test mid-pregnancy (fasting glucose ≥5.5 mmol/l and/or a 2 h glucose ≥8.5 mmol/l). Women who participated in the Target Trial with a singleton pregnancy and their infants were eligible for this study if they had completed a validated semi-quantitative food frequency questionnaire late in pregnancy (36 weeks gestation) [8] and data were available on maternal sociodemographics and health outcomes. The food frequency questionnaire consisted of 57 food items to assess maternal food intake over the past month. The health outcomes included were pre-eclampsia, onset of labour, caesarean section, postpartum haemorrhage, use of pharmaceuticals for GDM treatment, infant birthweight, gestational age at birth, shoulder dystocia, neonatal hypoglycaemia, neonatal hyperbilirubinaemia requiring treatment, neonatal respiratory support, and method of infant feeding at hospital discharge.

The method to assess maternal dietary adherence to the New Zealand Ministry of Health pregnancy guideline recommendations [9,10] has been previously reported [4]. Maternal dietary intake was measured using a food frequency questionnaire and analysed as a ratio against the 10 food-related dietary recommendations. Women who did not adhere to the recommendations achieved a minimum dietary adherence score of zero, while those who had complete adherence to the recommendations achieved a maximum score of 10. Adherence to the non-food-related recommendations—visit to a dietitian and appropriate weight gain—was measured as either yes or no. Adherence to the non-food-related recommendations was achieved if women visited a dietitian at least once after the diagnosis of GDM and achieved appropriate gestational weight gain according to BMI [11].

Participants were categorised into tertiles of low, moderate, or high dietary adherence based on dietary adherence scores. Sociodemographic characteristics of the women were summarised using descriptive statistics and compared using chi-square and ANOVA. Relationships between adherence to the recommendations and maternal and infant health outcomes were assessed using an odds ratio with 95% confidence intervals for categorical outcomes and t-tests for continuous outcomes. A two-sided *p*-value of <0.05 was considered statistically significant. Analyses were conducted using IBM SPSS Statistics 26 statistical software (version 26, Armonk, NY, USA).

## 3. Results

A total of 313 women with GDM and their infants were included. The median age of the women was 33.0 years (interquartile range 29.0–36.0 years); most were multiparous (55.6%) and did not smoke (91.7%) (Table 1). Most women were obese (56.6%) or overweight (32.9%); only 9.9% were normal weight, and a few were underweight (0.6%). Almost half were European (46.6%), followed by Asian (31.3%), Pacific (10.9%), and Māori (9.9%), with the remaining women of other ethnicities (1.3%). Over half of the women had a family history of diabetes (54.0%), although most had not had GDM previously (81.2%).

The mean dietary adherence score was 6.17/10 (standard deviation = 1.22, range = 2.35–9.49). Of the 313 women, 104 (33.2%) had low adherence, 105 (33.6%) moderate adherence and 104 (33.2%) high adherence to the food-related recommendations (Table 1). Most women (*n* = 269, 85.9%) adhered to the recommendation to visit a dietitian. Less than a third of women achieved the recommended gestational weight gain (*n* = 88, 28.1%), while similar numbers gained less (*n* = 110, 35.2%) or more weight (*n* = 115, 36.7%) than recommended.

Across the tertiles of dietary adherence, women had similar age, parity, BMI, smoking rates, ethnicity, socioeconomic deprivation, history of previous GDM and family history of diabetes. The sociodemographic profiles were similar for women who did or did not visit a dietitian and for women achieving below, appropriate or above the recommended weight gain (Table 1). Gestational age at trial entry was lower among women who visited a dietitian (median = 31.3, interquartile range = 29.9–32.3) compared to women who did not visit a dietitian (median = 32.0, interquartile range = 30.8–33.2) (Table 1).

Women with high dietary adherence compared to women with low adherence had lower rates of needing pharmacological treatment, oral hypoglycaemics and/or insulin for glucose control (63.5% versus 76.0%; OR = 0.55, 95%CI = 0.30–1.00, *p* = 0.05) (Table 2). High dietary adherence compared to low adherence was not associated with changes in the odds for use of oral hypoglycaemics alone, use of insulin alone, combination use of oral hypoglycaemics and insulin, hypoglycaemia, pre-eclampsia, induction of labour, elective or emergency caesarean section, postpartum haemorrhage, or method of infant feeding at hospital discharge (Table S1).

**Figure 1:**
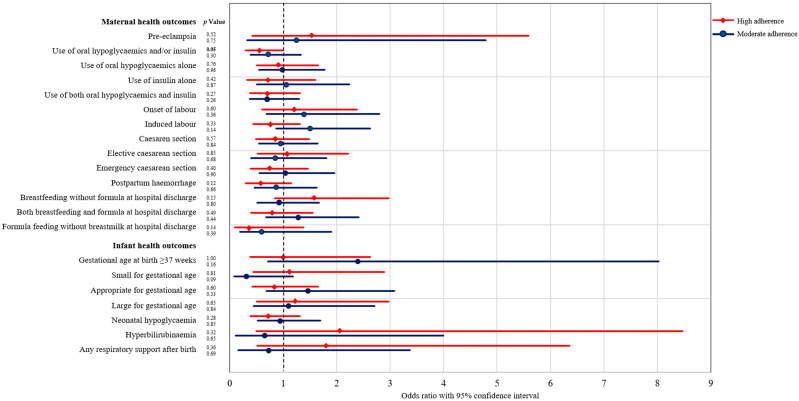
Forest plot for the odds of experiencing maternal and infant health outcomes in women with moderate (circles, blue) and high (diamonds, red) adherence to dietary recommendations compared with women with low adherence (reference group).

Moderate dietary adherence compared to low adherence among women was not associated with changes in the odds for use of oral hypoglycaemics alone, use of insulin alone, combination use of oral hypoglycaemics and/or insulin, hypoglycaemia, pre-eclampsia, induced or spontaneous labour, elective and emergency caesarean section, postpartum haemorrhage and breastfeeding, formula or combination feeding at hospital discharge (Figure 1).

Infants of women with high dietary adherence compared to infants of women with low adherence had similar rates of shoulder dystocia, preterm birth, hyperbilirubinaemia, need for respiratory support after birth, and mean birth weight and similar odds of neonatal hypoglycaemia, small-for-gestational-age (SGA), appropriate-for-gestational-age (AGA), and LGA (Figure 1).

Infants of women with moderate dietary adherence compared to infants of women with low adherence had a higher mean birthweight of 158 g (95%CI = 26–290, *p* = 0.02) (Table S2). Moderate adherence compared to low adherence was not associated with reduced odds of preterm birth, shoulder dystocia, neonatal hypoglycaemia, hyperbilirubinaemia, SGA, AGA, LGA, or need for respiratory support after birth (Figure 1).

**Figure 2:**
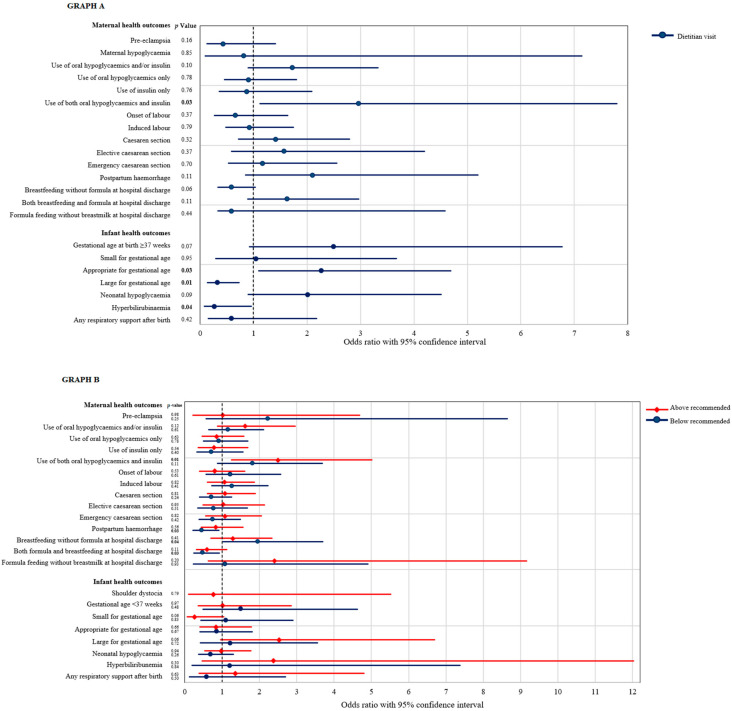
Forest plot for the odds of experiencing maternal and infant health outcomes in women who visited a dietitian ((**A**) circles, blue) compared with no dietitian visit ((**A**) reference group), and in women with low weight gain ((**B**) circles, blue) and high weight gain ((**B**) diamonds, red) compared with appropriate weight gain ((**B**) reference group).

Women who visited a dietitian compared to women who did not had higher rates of oral hypoglycaemics and insulin use (38.5% versus 19.3%; OR = 2.96, 95%CI = 1.12–7.80, *p* = 0.03) (Figure 2). Visiting a dietitian compared to no visit was not associated with reduced odds of pre-eclampsia, use of diet alone, use of oral hypoglycaemics alone or use of insulin alone, hypoglycaemia, caesarean section, postpartum haemorrhage, spontaneous and induced labour and breastfeeding, formula feeding and combination feeding at hospital discharge (Table S3).

Infants of women who visited a dietitian compared to infants of women who did not had more than twice the odds of being AGA (84.4% versus 70.5%, OR = 2.27, 95%CI = 1.10–4.69, *p* = 0.03) and lower odds of LGA (8.6% versus 22.7%, OR = 0.32, 95%CI = 0.14–0.73, *p* = 0.01) and developing neonatal hyperbilirubinaemia (2.6% versus 9.1%, OR = 0.27, 95%CI = 0.08–0.95, *p* = 0.04) (Table 2). Rates were similar for the other infant outcomes, comparing infants of women with GDM who visited a dietitian with those of women who did not, including shoulder dystocia, SGA, and neonatal respiratory support (Table S4).

Lower than recommended weight gain compared to appropriate weight gain was associated with reduced odds of postpartum haemorrhage (15.9% versus 29.4%; OR = 0.45, 95%CI = 0.23–0.91, *p* = 0.03), breastmilk and formula feeding at hospital discharge (17.3% versus 30.7%; OR = 0.47, 95%CI = 0.24–0.92, *p* = 0.03), and increased odds of fully breastfeeding at hospital discharge (79.1% versus 65.9%; OR = 1.96, 95%CI = 1.04–3.70, *p* = 0.04) but no difference in only formula feeding at hospital discharge (Figure 2). Achieving the recommended weight gain compared to weight gain below that recommended was not associated with altered odds for the remaining maternal health outcomes.

Women who gained more than the recommended weight compared to women who achieved appropriate weight gain had higher rates of receiving a combination of oral hypoglycaemics and insulin (43.0% versus 24.6%; OR = 2.51, 95%CI = 1.26–5.01, *p* = 0.01) (Figure 2), but similar rates of the remaining maternal health outcomes.

Achieving the appropriate gestational weight gain during pregnancy compared to gaining less than the recommended weight was not associated with reduced odds of preterm infant birth, SGA, AGA, LGA, hyperbilirubinaemia, neonatal hypoglycaemia, neonatal respiratory support and difference in mean infant birthweight (Table 2).

Infants of women who achieved appropriate weight gain compared to infants of women who gained more than the recommended weight had a lower mean birthweight of −175 g (95% CI = −38 to −311, *p* = 0.01) but similar rates of shoulder dystocia, infant birth size, pre-term birth, hypoglycaemia, hyperbilirubinemia, and neonatal respiratory support (Table 2).

## 4. Discussion

This study has demonstrated that different degrees of maternal adherence to the dietary recommendations within New Zealand clinical practice guidelines for the management of GDM are related to health outcomes for both the mother and infant.

High adherence to the food-related recommendations was associated with less oral hypoglycaemics and/or insulin use. Dietary intervention can normalise maternal glycaemic concentrations among most women with GDM by improving insulin sensitivity without the need for oral hypoglycaemics and insulin [12]. Oral hypoglycaemics and insulin use can place additional burdens on the mother, including adhering to a complex regimen of insulin injections and medication intake, gaining skills to self-administer the medication, and learning when to increase insulin dosage according to food consumption [13,14].

Moderate dietary adherence compared to low adherence was associated with a mean increase of 158 g in infant birthweight. Similarly, we found that gestational weight gain above that recommended compared to achieving appropriate weight gain was associated with a mean increase of 175 g in birthweight. Maternal dietary patterns characterised by high intakes of vegetables, fruit and dairy during pregnancy are associated with higher infant birthweights [15]. This increase in mean birthweight is of interest but may not be clinically significant as there was no difference in infant size categories (i.e., SGA, AGA or LGA) between the groups.

We observed decreasing rates of caesarean section, postpartum haemorrhage, neonatal hypoglycaemia, and formula feeding at hospital discharge as dietary adherence to the food-related recommendations increased across tertiles. While similar trends have not been observed in the literature, some studies have reported the association between maternal diets high in vegetables and the decreased incidence of these health outcomes [16,17]. The patterns we found may indicate a positive trend between increasing dietary adherence and a lower incidence of these adverse health outcomes.

Visiting a dietitian was associated with increased insulin and oral hypoglycaemic use, which has been observed in previous studies [18]. The New Zealand guideline recommends that women should visit a dietitian after the diagnosis of GDM to help manage their blood glucose concentrations by dietary interventions [10]. However, a survey of dietitians in New Zealand reported that 89% of referrals of women with GDM occurred due to poor glycaemic control, and over 50% of dietitians saw women with GDM after the commencement of oral hypoglycaemics or insulin [19]. Regulating blood glucose concentrations via dietitian guidance tailored to individual nutritional needs based on lifestyle and pharmacological use may improve the management of GDM [20].

Visiting a dietitian was associated with lower odds of women having an infant born LGA and needing treatment for hyperbilirubinemia. Effective dietary interventions in combination with appropriate nutrition counselling techniques, skills and knowledge provided by a dietitian have been shown to help normalise blood glucose concentrations [20]. In a woman with GDM, high blood glucose concentrations increase fetal insulin production and can result in fetal overgrowth, leading to an LGA infant [21]. LGA infants have higher haematocrits than AGA infants, resulting in an increase in haeme turnover and an increased risk of neonatal hyperbilirubinaemia [22]. LGA infants also have a greater risk of being born by operative delivery and having birth injuries and nerve damage compared to AGA infants. In childhood and adulthood, they have an increased risk of being overweight and obese, having hypertension, and metabolic syndrome [23,24,25].

Our results showed that appropriate weight gain compared to above-recommended weight gain was associated with less insulin and oral hypoglycaemic use. Excessive gestational weight gain is associated with decreased insulin sensitivity, which results in an increase in blood glucose concentrations [26,27]. Oral hypoglycaemic drugs and insulin are used to improve glycaemic control when dietary intervention alone is insufficient to control maternal hyperglycaemia in women with GDM.

Breastmilk is the preferred method of infant feeding as it is the best source of nutrition, contains antibodies and adapts to the need of the infant [28]. We found that achieving appropriate gestational weight gain compared to weight gain below the recommended was associated with lower odds of breastfeeding at hospital discharge. In contrast, a meta-analysis of five cohort studies involving 3506 pregnant women, including those with GDM, found that women who did not gain the recommended gestational weight compared to women with appropriate weight gain were 27% less likely to initiate breastfeeding [29]. However, other factors that can influence breastfeeding initiation include unplanned pregnancy, failure to attach, insufficient milk supply and maternal attitude towards breastfeeding, none of which were measured in our cohort [30]. Planned pregnancy has been reported to be an indicator of lower gestational weight gain compared to unplanned pregnancy, [31] and planned pregnancy is also associated with higher breastfeeding rates in New Zealand [30].

To our knowledge, this cohort study is one of the first to explore the associations between adherence to dietary recommendations for the management of GDM and maternal and infant health. Women who participated in the Target Trial were recruited from ten hospitals throughout New Zealand so that the cohort is nationally representative. The results of this study may be generalised to other populations of pregnant women with GDM as our participants were multi-ethnic and included primipara and multipara. Furthermore, we measured maternal adherence to dietary intervention, which is the recommended treatment for GDM globally. Our study had the following limitations: assessment of maternal dietary intake relied on self-report, which may have introduced recall bias; the observational design of the study could have introduced bias from possible confounding factors; the lack of expected associations found could be due to the limited power in our study and the low incidence of some outcomes such as maternal hypoglycaemia and infant shoulder dystocia, leading to wide confidence intervals.

## 5. Conclusions

In conclusion, improving dietary adherence to dietary- and non-dietary-related recommendations among women with GDM is associated with improved maternal and infant health. Interventions to improve dietary adherence may result in better health outcomes.

## Figures and Tables

**Table 1:** Baseline characteristics of 313 participants included from the TARGET Trial.

Maternal Sociodemographics	Adherence to Food-Related Recommendations				Adherence to Non-Food-Related Recommendations						
	Dietary Adherence ^2^ *n* (%)				Visited A Dietitian *n* (%)			Gestational Weight Gain *n* (%)			
	Low (T1) *n* = 104	Moderate (T2) *n* = 105	High (T3) *n* = 104	*p*-Value	Yes *n* = 269	No *n* = 44	*p* Value	Below Recommended *n* = 110	Recommended *n* = 88	Above Recommended *n* = 115	*p*-Value
Age in years ^1^	33.0 (29.0, 35.0)	32.0 (29.0, 36.0)	33.0 (30.0, 36.0)	0.42	33.0 (29.0, 36.0)	34.0 (30.0, 37.0)	0.66	33.0 (29.0, 36.0)	34.0 (30.3, 37.0)	32.0 (30.0, 35.0)	0.10
≤20 to 24 years	5 (4.8)	3 (2.9)	5 (4.8)		12 (4.5)	1 (2.3)		2 (1.8)	4 (4.5)	7 (6.1)	
25 to 29 years	25 (24.0)	25 (23.8)	16 (15.4)		59 (21.9)	7 (15.9)		30 (27.3)	15 (17.0)	21 (18.3)	
30 to 34 years	34 (32.7)	34 (32.4)	46 (44.2)		99 (36.8)	15 (34.1)		38 (34.5)	26 (29.5)	50 (43.5)	
35 to 39 years	31 (29.8)	35 (33.3)	25 (24.0)		74 (27.5)	17 (38.6)		28 (25.5)	33 (37.5)	30 (26.1)	
≥40 years	9 (8.7)	8 (7.6)	12 (11.5)		25 (9.3)	4 (9.1)		12 (10.9)	10 (11.4)	7 (6.1)	
Ethnicity				0.97			0.16				0.25
European	45 (43.3)	52 (49.5)	49 (47.1)		121 (45.0)	25 (56.8)		49 (44.5)	44 (50.0)	53 (46.1)	
Asian	37 (35.6)	31 (29.5)	30 (28.8)		86 (32.0)	12 (27.3)		35 (31.8)	32 (36.4)	31 (27.0)	
Pacific People	12 (11.5)	10 (9.5)	12 (11.5)		33 (12.3)	1 (2.3)		10 (9.1)	9 (10.2)	15 (13.0)	
Māori	9 (8.7)	11 (10.5)	11 (10.6)		26 (9.7)	5 (11.4)		14 (12.7)	2 (2.3)	15 (13.0)	
Other	1 (1.0)	1 (1.0)	2 (1.9)		3 (1.1)	1 (2.3)		2 (1.8)	1 (1.1)	1 (0.9)	
Socioeconomic deprivation index				0.35			0.35				0.07
1 to 2 (least deprived)	16 (15.4)	26 (24.8)	26 (25.0)		54 (20.1)	14 (31.8)		17 (15.5)	26 (38.2)	25 (21.7)	
3 to 4	21 (20.2)	15 (14.3)	10 (9.6)		40 (14.9)	6 (13.6)		16 (14.5)	8 (17.4)	22 (19.1)	
5 to 6	12 (11.5)	14 (13.3)	17 (16.3)		40 (14.9)	3 (6.8)		14 (12.7)	8 (18.6)	21 (18.3)	
7 to 8	23 (22.1)	18 (17.1)	17 (16.3)		49 (18.2)	9 (20.5)		22 (20.0)	18 (31.0)	18 (15.7)	
9 to 10 (most deprived)	32 (30.8)	32 (30.5)	34 (32.7)		86 (32.0)	12 (27.3)		41 (37.3)	28 (28.6)	29 (25.2)	
Previous GDM	19 (18.3)	13 (12.5)	25 (24.5)	0.08	51 (19.2)	6 (13.6)	0.38	21 (19.3)	14 (16.1)	22 (19.3)	0.81
Primiparous	48 (46.2)	52 (49.5)	39 (37.5)	0.20	120 (44.6)	19 (43.2)	0.86	47 (42.7)	43 (48.9)	49 (42.6)	0.61
BMI				0.63			0.25				0.10
Underweight	1 (1.0)	1 (1.0)	0 (0.00)		2 (0.7)	0 (0.0)		1 (0.9)	1 (1.1)	0 (0.0)	
Normal	8 (7.7)	11 (10.5)	12 (11.5)		30 (11.2)	1 (2.3)		17 (15.5)	9 (10.2)	5 (4.3)	
Overweight	29 (27.9)	37 (35.2)	37 (35.6)		86 (32.0)	17 (38.6)		32 (29.1)	33 (37.5)	38 (33.0)	
Obese	66 (63.5)	56 (53.3)	55 (52.9)		151 (56.1)	26 (59.1)		60 (54.5)	45 (51.1)	72 (62.6)	
Family history of diabetes	53 (53.0)	54 (54.5)	62 (61.4)	0.44	139 (54.1)	30 (69.8)	0.06	57 (54.3)	52 (61.2)	60 (54.5)	0.57
Smoking at trial entry	11 (10.6)	9 (8.7)	6 (5.8)	0.45	24 (9.0)	2 (4.5)	0.55	8 (7.3)	6 (6.8)	12 (10.4)	0.59
Gestational age at trial entry ^1^	31.4 (30.1, 32.4)	31.4 (30.3, 32.5)	31.3 (29.8, 32.3)	0.78	31.3 (29.9, 32.3)	32.0 (30.8, 33.2)	0.01 *	31.2 (30.0, 32.3)	31.6 (30.0, 32.4)	31.4 (30.0, 32.6)	0.86

Figures are numbers (%) or ^1^ median (interquartile range). ^2^ Tertiles: T1: dietary score 0 to 5.6, T2: dietary score >5.6 to 6.7, T3: dietary score >6.7 to 10. BMI, body mass index. GDM, gestational diabetes mellitus. * *p*-value with an asterisk indicates statistical significance <0.05.

**Table 2:** Dietary adherence to the recommendations and frequency of maternal and infant health outcomes.

Health Outcomes	Adherence to Food-Related Recommendations			Adherence to Non-Food-Related Recommendations				
	Dietary Adherence ^2^ *n* (%)			Visited a Dietitian *n* (%)		Gestational Weight Gain *n* (%)		
	Low (T1) *n* = 104	Moderate (T2) *n* = 105	High (T3) *n* = 104	Yes *n* = 269	No *n* = 44	Below Recommended *n* = 110	Recommended *n* = 88	Above Recommended *n* = 115
Maternal health outcomes								
Pre-eclampsia	4 (3.8)	5 (4.8)	6 (5.8)	11 (4.1)	4 (9.1)	8 (7.3)	3 (3.4)	4 (3.5)
Maternal hypoglycaemia	1 (1.0)	4 (3.8)	1 (1.0)	5 (1.9)	1 (2.3)	4 (3.6)	0 (0.0)	2 (1.7)
Pharmacological use								
Diet alone	25 (24.0)	32 (30.5)	38 (36.5)	77 (28.6)	18 (40.9)	35 (31.8)	31 (35.2)	29 (25.2)
Use of oral hypoglycaemics or/and insulin	79 (76.0)	73 (69.5)	66 (63.5)	192 (71.4)	26 (59.1)	75 (68.2)	57 (64.8)	86 (74.8)
Use of oral hypoglycaemics alone	32 (40.5)	32 (43.8)	30 (45.5)	80 (41.7)	14 (53.8)	33 (44.0)	28 (49.1)	33 (38.4)
Use of insulin alone	16 (20.3)	17 (23.3)	12 (18.2)	38 (19.8)	7 (26.9)	14 (18.7)	15 (26.3)	16 (18.6)
Use of oral hypoglycaemics and insulin	31 (39.2)	24 (32.9)	24 (36.4)	74 (38.5)	5 (19.3)	28 (37.3)	14 (24.6)	37 (43.0)
Labour	82 (78.8)	88 (83.8)	85 (81.7)	217 (80.7)	38 (86.4)	93 (84.5)	72 (81.8)	90 (78.3)
Spontaneous labour	26 (31.7)	21 (23.9)	36 (42.4)	70 (32.3)	13 (34.2)	29 (31.2)	26 (36.1)	28 (31.1)
Induced labour	56 (68.3)	67 (76.1)	49 (57.6)	147 (67.7)	25 (65.8)	64 (68.8)	46 (63.9)	62 (68.9)
Caesarean section	42 (40.4)	41 (39.0)	38 (36.5)	107 (39.8)	14 (31.8)	36 (32.7)	36 (40.9)	49 (42.6)
Elective caesarean section	17 (40.5)	15 (36.6)	18 (47.4)	45 (42.1)	5 (35.7)	15 (41.7)	15 (41.7)	20 (40.8)
Emergency caesarean section	25 (59.5)	26 (63.4)	20 (52.6)	62 (57.9)	9 (64.3)	21 (58.3)	21 (58.3)	29 (59.2)
Postpartum haemorrhage	28 (27.2)	25 (24.5)	18 (18.0)	65 (24.9)	6 (13.6)	17 (15.9)	25 (29.4)	29 (25.7)
Feeding at hospital discharge								
Breastmilk without formula	73 (70.2)	72 (68.6)	82 (78.8)	196 (72.9)	31 (70.5)	87 (79.1)	58 (65.9)	82 (71.3)
Both formula and breastmilk	23 (22.1)	28 (26.7)	19 (18.3)	60 (22.3)	10 (22.7)	19 (17.3)	27 (30.7)	24 (20.9)
Formula without breastmilk	8 (7.7)	5 (4.8)	3 (2.9)	13 (4.8)	3 (6.8)	4 (3.6)	3 (3.4)	9 (7.8)
Infant health outcomes								
Shoulder dystocia	1 (1.0)	1 (1.0)	2 (1.9)	4 (1.5)	0 (0.0)	0 (0.0)	2 (2.3)	2 (1.7)
Birthweight in grams, mean (SD) ^1^	3227.6 (493.1)	3385.5 (475.0)	3322.6 (494.7)	3293.2 (479.4)	3427.7 (543.0)	3211.6 (469.6)	3267.6 (479.8)	3442.40 (493.2)
Gestational age at birth <37 weeks	9 (8.7)	4 (3.8)	9 (8.7)	16 (5.9)	6 (13.6)	6 (5.5)	7 (8.0)	9 (7.8)
Gestational age at birth ≥37 weeks	95 (91.3)	101 (96.2)	95 (91.3)	253 (94.1)	38 (86.4)	104 (94.5)	81 (92.0)	106 (92.2)
SGA	9 (8.7)	3 (2.9)	10 (9.6)	19 (7.1)	3 (6.8)	11 (10.0)	8 (9.1)	3 (2.6)
AGA	85 (81.7)	91 (86.7)	82 (78.8)	227 (84.4)	31 (70.5)	90 (81.8)	74 (84.1)	94 (81.7)
LGA	10 (9.6)	11 (10.5)	12 (11.5)	23 (8.6)	10 (22.7)	9 (8.2)	6 (6.8)	18 (15.7)
Neonatal hypoglycaemia	33 (31.7)	32 (30.5)	26 (25.0)	83 (30.9)	8 (18.2)	27 (24.5)	28 (31.8)	36 (31.3)
Hyperbilirubinaemia	3 (2.9)	2 (1.9)	6 (5.8)	7 (2.6)	4 (9.1)	3 (2.7)	2 (2.3)	6 (5.2)
Any respiratory support after birth	4 (3.8)	3 (2.9)	7 (6.7)	11 (4.1)	3 (6.8)	3 (2.7)	4 (4.5)	7 (6.1)

Figures are numbers (%) or ^1^ mean (standard deviation). ^2^ Tertiles: T1: dietary score 0 to 5.6, T2: dietary score >5.6 to 6.7, T3: dietary score >6.7 to 10. SD: standard deviation; SGA: small-for-gestational-age; AGA: appropriate-for-gestational-age; LGA: large-for-gestational-age.

## Data Availability

Published data are available to approved researchers under the data-sharing arrangements provided by the Maternal and Perinatal Central Coordinating Research Hub (CCRH), based at the Liggins Institute, University of Auckland (https://wiki.auckland.ac.nz/researchhub, accessed on 15 March 2022). Data access requests are to be submitted to Data Access Committee via researchhub@auckland.ac.nz. Data will be shared with researchers who provide a methodologically sound proposal and have appropriate ethical and institutional approval. Researchers must sign and adhere to the Data Access Agreement that includes a commitment to using the data only for the specified proposal, to store data securely and to destroy or return the data after completion of the project. The CCRH reserves the right to charge a fee to cover the costs of making data available, if required.

## Supplementary Materials

The following supporting information can be downloaded at: https://www.mdpi.com/article/10.3390/nu14061274/s1, Table S1: Association of maternal dietary adherence to the food-related recommendations on maternal health outcomes; Table S2: Association of maternal dietary adherence to the food-related recommendations on maternal health outcomes; Table S3. Association of maternal dietary adherence to the non-food-related recommendations on maternal health outcomes; Table S4. Association of maternal dietary adherence to the non-food-related recommendations on infant health outcomes.
