# Supplementary material for: Adherence to Clinical Practice Guideline Recommendations in Women with Gestational Diabetes and Associations with Maternal and Infant Health—A Cohort Study
Source: Nutrients. 2022 Mar 17;14(6):1274. doi: 10.3390/nu14061274 (PMC8949953; doi:10.3390/nu14061274)
Supplement: Supplementary file 1 [file nutrients-14-01274-s001.zip › nutrients-1610506-supplementary.pdf]

Supplementary Tables

Results

Table S1. Association of maternal dietary adherence to the food-related recommendations on maternal health outcomes.

Maternal health outcomes	Dietary adherence [1]										
	Low (T1) [2]	Moderate (T2)					High (T3)				
	N (%)	N (%)	P	OR	95% L	95% U	N (%)	P	OR	95% L	95% U
Pre-eclampsia	4 (3.8)	5 (4.8)	0.75	1.25	0.33	4.79	6 (5.8)	0.52	1.53	0.42	5.59
No pre-eclampsia	100 (96.2)	100 (95.2)	0.75	0.80	0.21	3.07	98 (94.2)	0.52	0.65	0.18	2.39
Maternal hypoglycaemia	1 (1.0)	4 (3.8)	0.21	4.08	0.45	37.13	1 (1.0)	1.00	1.00	0.06	16.20
No maternal hypoglycaemia	103 (99.0)	101 (96.2)	0.21	0.25	0.03	2.23	103 (99.0)	1.00	1.00	0.06	16.20
Pharmacological use											
Diet alone	25 (24.0)	32 (30.5)	0.30	1.39	0.75	2.56	38 (36.5)	0.05	1.82	1.00	3.32
Use of either oral hypoglycaemics or/and insulin	79 (76.0)	73 (69.5)	0.30	0.72	0.39	1.33	66 (63.5)	0.05	0.55	0.30	1.00
Use of oral hypoglycaemics only	32 (40.5)	32 (43.8)	0.96	0.99	0.55	1.78	30 (45.5)	0.76	0.91	0.50	1.65
Use of insulin only	16 (20.3)	17 (23.3)	0.87	1.06	0.51	2.24	12 (18.2)	0.42	0.72	0.32	1.60
Use of oral hypoglycaemics and insulin	31 (39.2)	24 (32.9)	0.26	0.70	0.38	1.30	24 (36.4)	0.27	0.71	0.38	1.31
Onset of labour											
No labour	22 (21.2)	17 (16.2)	0.36	0.72	0.36	1.45	19 (18.3)	0.60	0.83	0.42	1.65
Labour	82 (78.8)	88 (83.8)	0.36	1.39	0.69	2.80	85 (81.7)	0.60	1.20	0.61	2.38
Spontaneous labour	26 (31.7)	21 (23.9)	0.39	0.75	0.39	1.44	36 (42.4)	0.13	1.59	0.87	2.89
Induced labour	56 (68.3)	67 (76.1)	0.14	1.51	0.87	2.63	49 (57.6)	0.33	0.76	0.44	1.32
Caesarean section											
No caesarean section	62 (59.6)	64 (61.0)	0.84	1.06	0.61	1.84	66 (63.5)	0.57	1.18	0.67	2.06
Caesarean section	42 (40.4)	41 (39.0)	0.84	0.95	0.54	1.65	38 (36.5)	0.57	0.85	0.49	1.49
Elective caesarean section	17 (40.5)	15 (36.6)	0.68	0.85	0.40	1.81	18 (47.4)	0.85	1.07	0.52	2.22
Emergency caesarean section	25 (59.5)	26 (63.4)	0.90	1.04	0.55	1.96	20 (52.6)	0.40	0.75	0.39	1.46
Postpartum haemorrhage	28 (27.2)	25 (24.5)	0.66	0.87	0.47	1.63	18 (18.0)	0.12	0.59	0.30	1.15
No postpartum haemorrhage	75 (72.8)	77 (75.5)	0.66	1.15	0.62	2.15	82 (82.0)	0.12	1.70	0.87	3.32

Feeding at hospital discharge											
Breastmilk without formula	73 (70.2)	72 (68.6)	0.80	0.93	0.51	1.67	82 (78.8)	0.15	1.58	0.84	2.98
Formula without breastmilk	8 (7.7)	5 (4.8)	0.39	0.60	0.19	1.90	3 (2.9)	0.14	0.36	0.09	1.38
Both formula and breastmilk	23 (22.1)	28 (26.7)	0.44	1.28	0.68	2.41	19 (18.3)	0.49	0.79	0.40	1.55

Figures are numbers (%). [1]Tertiles: T1, dietary score 0 to 5.6. T2, dietary score >5.6 to 6.7. T3, dietary score >6.7 to 10. [2]Low adherence (T1) is reference group.

Table S2. Association of maternal dietary adherence to the food-related recommendations on infant health outcomes.

Infant health outcomes	Dietary adherence[2]										
	Low (T1)[3]	Moderate (T2)					High (T3)				
	N (%)	N (%)	P	OR	95% L	95% U	N (%)	P	OR	95% L	95% U
Shoulder dystocia	1 (1.0)	1 (1.0)	1.00	0.99	0.06	16.05	2 (1.9)	0.57	2.02	0.18	22.62
No shoulder dystocia	103 (99.0)	103 (99.0)	1.00	1.01	0.06	16.36	102 (98.1)	0.57	0.50	0.04	5.55
Birthweight in grams, mean (SD)[1]	3227.6 (493.1)	3385.5 (475.0)	0.02	Mean difference: 157.95	25.91	290.00	3322.6 (494.7)	0.17	Mean difference: 95.02	-40.01	230.05
Gestational age at birth <37 weeks	9 (8.7)	4 (3.8)	0.16	0.42	0.13	1.40	9 (8.7)	1.00	1.00	0.38	2.63
Gestational age at birth ≥37 weeks	95 (91.3)	101 (96.2)	0.16	2.39	0.71	8.03	95 (91.3)	1.00	1.00	0.38	2.63
SGA	9 (8.7)	3 (2.9)	0.09	0.31	0.08	1.18	10 (9.6)	0.81	1.12	0.44	2.89
AGA	85 (81.7)	91 (86.7)	0.33	1.45	0.69	3.08	82 (78.8)	0.60	0.83	0.42	1.65
LGA	10 (9.6)	11 (10.5)	0.84	1.10	0.45	2.71	12 (11.5)	0.65	1.23	0.51	2.98
Neonatal hypoglycaemia	33 (31.7)	32 (30.5)	0.85	0.94	0.53	1.69	26 (25.0)	0.28	0.72	0.39	1.32
No neonatal hypoglycaemia	71 (68.3)	73 (69.5)	0.85	1.06	0.59	1.91	78 (75.0)	0.28	1.39	0.76	2.56
Hyperbilirubinaemia	3 (2.9)	2 (1.9)	0.65	0.65	0.11	4.00	6 (5.8)	0.32	2.06	0.50	8.47
No hyperbilirubinaemia	101 (97.1)	103 (98.1)	0.65	1.53	0.25	9.35	98 (94.2)	0.32	0.49	0.12	1.99
Any respiratory support after birth	4 (3.8)	3 (2.9)	0.69	0.74	0.16	3.37	7 (6.7)	0.36	1.80	0.51	6.36
No use of respiratory support after birth	100 (96.2)	102 (97.1)	0.69	1.36	0.30	6.23	97 (93.3)	0.36	0.55	0.16	1.95

Figures are numbers (%) or [1]mean (standard deviation). [2]Tertiles: T1, dietary score 0 to 5.6. T2, dietary score >5.6 to 6.7. T3, dietary score >6.7 to 10. [3]Low adherence (T1) is reference group. SGA: small-for-gestational-age; AGA: appropriate-for-gestational-age; LGA: large-for-gestational-age

Table S3. Association of maternal dietary adherence to the non-food-related recommendations on maternal health outcomes.

Maternal health outcomes	Adherence to non-food-related recommendations										
	Visited a dietitian				Gestational weight gain						
	Yes			No[1]	Below recommended			Recommended[2]	Above recommended		
	N (%)	P	OR, 95% CI	N (%)	N (%)	P	OR, 95% CI	N (%)	N (%)	P	OR, 95% CI
Pre-eclampsia	11 (4.1)	0.16	0.43, 0.13-1.40	4 (9.1)	8 (7.3)	0.25	2.22, 0.57-8.64	3 (3.4)	4 (3.5)	0.98	1.02, 0.22-4.68
No pre-eclampsia	258 (95.9)	0.16	2.35, 0.71-7.72	40 (90.9)	102 (92.7)	0.25	0.45, 0.12-1.75	85 (96.6)	111 (96.5)	0.98	0.98, 0.21-4.49
Maternal hypoglycaemia	5 (1.9)	0.85	0.81, 0.09-7.14	1 (2.3)	4 (3.6)	NC	NC	0 (0.0)	2 (1.7)	NC	NC
No maternal hypoglycaemia	264 (98.1)	0.85	1.23, 0.14-10.77	43 (97.7)	106 (96.4)	NC	NC	88 (100.0)	113 (98.3)	NC	NC
Pharmacological use											
Diet alone	77 (28.6)	0.10	0.58, 0.30-1.12	18 (40.9)	35 (31.8)	0.61	0.86, 0.47-1.55	31 (35.2)	29 (25.2)	0.12	0.62, 0.34-1.14
Use of either oral hypoglycaemics or/and insulin	192 (71.4)	0.10	1.73, 0.90-3.33	26 (59.1)	75 (68.2)	0.61	1.17, 0.64-2.11	57 (64.8)	86 (74.8)	0.12	1.61, 0.88-2.96
Use of oral hypoglycaemics only	80 (41.7)	0.78	0.91, 0.46-1.80	14 (53.8)	33 (44.0)	0.78	0.92, 0.50-1.68	28 (49.1)	33 (38.4)	0.63	0.86, 0.47-1.58
Use of insulin only	38 (19.8)	0.76	0.87, 0.36-2.09	7 (26.9)	14 (18.7)	0.40	0.71, 0.32-1.56	15 (26.3)	16 (18.6)	0.54	0.79, 0.37-1.69
Use of oral hypoglycaemics and insulin	74 (38.5)	0.03	2.96, 1.12-7.80	5 (19.3)	28 (37.3)	0.11	1.81, 0.88-3.69	14 (24.6)	37 (43.0)	0.01	2.51, 1.26-5.01
Onset of labour											
No labour	52 (19.3)	0.37	1.52, 0.61-3.78	6 (13.6)	17 (15.5)	0.61	0.82, 0.39-1.74	16 (18.2)	25 (21.7)	0.53	1.25, 0.62-2.52
Labour	217 (80.7)	0.37	0.66, 0.27-1.64	38 (86.4)	93 (84.5)	0.61	1.22, 0.58-2.57	72 (81.8)	90 (78.3)	0.53	0.80, 0.40-1.61
Spontaneous labour	70 (32.3)	0.62	0.84, 0.42-1.69	13 (34.2)	29 (31.2)	0.62	0.85, 0.46-1.59	26 (36.1)	28 (31.1)	0.41	0.77, 0.41-1.43
Induced labour	147 (67.7)	0.79	0.92, 0.48-1.74	25 (65.8)	64 (68.8)	0.41	1.27, 0.72-2.23	46 (63.9)	62 (68.9)	0.82	1.07, 0.61-1.86
Caesarean section											
No caesarean section	162 (60.2)	0.32	0.71, 0.36-1.39	30 (68.2)	74 (67.3)	0.24	1.42, 0.80-2.55	52 (59.1)	66 (57.4)	0.81	0.93, 0.53-1.64
Caesarean section	107 (39.8)	0.32	1.42, 0.72-2.79	14 (31.8)	36 (32.7)	0.24	0.70, 0.39-1.26	36 (40.9)	49 (42.6)	0.81	1.07, 0.61-1.88
Elective caesarean section	45 (42.1)	0.37	1.57, 0.59-4.19	5 (35.7)	15 (41.7)	0.51	0.77, 0.35-1.67	15 (41.7)	20 (40.8)	0.95	1.03, 0.49-2.14
Emergency caesarean section	62 (57.9)	0.70	1.17, 0.53-2.56	9 (64.3)	21 (58.3)	0.42	0.75, 0.30-1.49	21 (58.3)	29 (59.2)	0.82	1.08, 0.56-2.05

Postpartum haemorrhage	65 (24.9)	0.11	2.10, 0.85-5.20	6 (13.6)	17 (15.9)	0.03	0.45, 0.23-0.91	25 (29.4)	29 (25.7)	0.56	0.83, 0.44-1.55
No postpartum haemorrhage	196 (75.1)	0.11	0.48, 0.19-1.18	38 (86.4)	90 (84.1)	0.03	2.21, 1.10-4.43	60 (70.6)	84 (74.3)	0.56	1.21, 0.64-2.26
Feeding at hospital discharge											
Formula without breastmilk	196 (72.9)	0.44	1.54, 0.52-4.58	31 (70.5)	4 (3.6)	0.93	1.07, 0.23-4.91	3 (3.4)	9 (7.8)	0.20	2.41, 0.63-9.16
Breastmilk without formula	13 (4.8)	0.06	0.58, 0.33-1.03	3 (6.8)	87 (79.1)	0.04	1.96, 1.04-3.70	58 (65.9)	82 (71.3)	0.41	1.29, 0.71-2.34
Both formula and breastmilk	60 (22.3)	0.11	1.63, 0.89-2.97	10 (22.7)	19 (17.3)	0.03	0.47, 0.24-0.92	27 (30.7)	24 (20.9)	0.11	0.60, 0.32-1.13

Figures are numbers (%). [1]No visit to the dietitian is the reference group. [2]Achieving recommended weight gain is the reference group. NC: not calculable.

Table S4. Association of maternal dietary adherence to the non-food-related recommendations on infant health outcomes.

Infant health outcomes	Adherence to non-food-related recommendations										
	Visited a dietitian				Gestational weight gain						
	Yes			No [2]	Below recommended			Recommended[3]	Above recommended		
	N (%)	P	OR, 95% CI	N (%)	N (%)	P	OR, 95% CI	N (%)	N (%)	P	OR, 95% CI
Shoulder dystocia	4 (1.5)	NC	NC	0 (0.0)	0 (0.0)	NC	NC	2 (50.0)	2 (50.0)	0.79	0.76, 0.11-5.51
No shoulder dystocia	265 (98.5)	NC	NC	44 (100.0)	110 (100.0)	NC	NC	86 (97.7)	113 (98.3)		
Birthweight in grams [1]	3293.2 (479.4)	0.09	Mean difference: -134.44, CI: -290.90, 22.01	3427.7 (543.0)	3211.6 (469.6)	0.41	Mean difference: -56.00, CI: -77.75-189.75	3267.6 (479.8)	3442.40 (493.2)	0.01	Mean difference: 174.84, CI: 38.71-310.97
Gestational age at birth <37 weeks	16 (5.9)	0.07	0.40, 0.15-1.09	6 (13.6)	6 (5.5)	0.48	0.67, 0.22-2.06	7 (8.0)	9 (7.8)	0.97	0.98, 0.35-2.75
Gestational age at birth ≥37 weeks	253 (94.1)	0.07	2.50, 0.92-6.78	38 (86.4)	104 (94.5)	0.48	1.50, 0.49-4.63	81 (92.0)	106 (92.2)	0.97	1.02, 0.36-2.85
SGA	19 (7.1)	0.95	1.04, 0.29-3.67	3 (6.8)	11 (10.0)	0.83	1.11, 0.43-2.89	8 (9.1)	3 (2.6)	0.06	0.27, 0.07-1.04
AGA	227 (84.4)	0.03	2.27, 1.10-4.69	31 (70.5)	90 (81.8)	0.67	0.85, 0.40-1.80	74 (84.1)	94 (81.7)	0.66	0.85, 0.40-1.78
LGA	23 (8.6)	0.01	0.32, 0.14-0.73	10 (22.7)	9 (8.2)	0.72	1.22, 0.42-3.56	6 (6.8)	18 (15.7)	0.06	2.54, 0.96-6.69
Neonatal hypoglycaemia	83 (30.9)	0.09	2.01, 0.90-4.51	8 (18.2)	27 (24.5)	0.26	0.70, 0.37-1.30	28 (31.8)	36 (31.3)	0.94	0.98, 0.54-1.77
No neonatal hypoglycaemia	186 (69.1)	0.09	0.50, 0.22-1.12	36 (81.8)	83 (75.5)	0.26	1.44, 0.77-2.68	60 (68.2)	79 (68.7)	0.94	1.02, 0.56-1.86
Hyperbilirubinaemia	7 (2.6)	0.04	0.27, 0.08-0.95	4 (9.1)	3 (2.7)	0.84	1.21, 0.20-7.38	2 (2.3)	6 (5.2)	0.30	2.37, 0.47-12.02
No hyperbilirubinaemia	262 (97.4)	0.04	3.74, 1.05-13.36	40 (90.9)	107 (97.3)	0.84	0.83, 0.14-5.08	86 (97.7)	109 (94.8)	0.30	0.42, 0.08-2.15
Any respiratory support after birth	11 (4.1)	0.42	0.58, 0.16-2.18	3 (6.8)	3 (2.7)	0.50	0.59, 0.13-2.70	4 (4.5)	7 (6.1)	0.63	1.36, 0.39-4.80
No use of respiratory support after birth	258 (95.9)	0.42	1.72, 0.46-6.41	41 (93.2)	107 (97.3)	0.50	1.70, 0.37-7.80	84 (95.5)	108 (93.9)	0.63	0.74, 0.21-2.59

Figures are numbers (%) or [1] mean (standard deviation). [2] No dietitian visit is the reference group. [3] Achieving recommended weight gain is the reference group. NC: not calculable. SGA: small-for-gestational-age; AGA: appropriate-for-gestational-age; LGA: large-for-gestational-age
